# The Infectious Bronchitis Coronavirus Pneumonia Model Presenting a Novel Insight for the SARS-CoV-2 Dissemination Route

**DOI:** 10.3390/vetsci8100239

**Published:** 2021-10-18

**Authors:** Ekaterina Nefedova, Vyacheslav Koptev, Anna S. Bobikova, Viktoria Cherepushkina, Tatyana Mironova, Vasily Afonyushkin, Nikolai Shkil, Nikolai Donchenko, Yulia Kozlova, Natalia Sigareva, Natalia Davidova, Nina Bogdanchikova, Alexey Pestryakov, Yanis Toledano-Magaña

**Affiliations:** 1 Siberian Federal Scientific Center of Agro-BioTechnologies of Russian Academy of Sciences, Novosibirsk Region, Novosibirsk District, 630501 Krasnoobsk, Russia; filll555@mail.ru (E.N.); kastrolog@mail.ru (V.K.); bobikova.anna97@gmail.com (A.S.B.); vicky88@bk.ru (V.C.); mironova.tanya1994@mail.ru (T.M.); lisocim@mail.ru (V.A.); nicola07@mail.ru (N.S.); tbc2009@yandex.ru (N.D.); ulona79@mail.ru (N.D.); 2 Molecular Biology Department, Federal State Budgetary Educational Institution Higher Education Novosibirsk State Agrarian University, 630090 Novosibirsk, Russia; natalias72@mail.ru; 3 Institute of Chemical Biology and Fundamental Medicine SB RAS, 630090 Novosibirsk, Russia; ramira_@bk.ru; 4 Centro de Nanociencias y Nanotecnología, Universidad Nacional Autónoma de México, Ensenada 22860, BC, Mexico; 5 Research School of Chemistry & Applied Biomedical Sciences, National Research Tomsk Polytechnic University, 634050 Tomsk, Russia; pestryakov2005@yandex.ru; 6 Escuela de Ciencias de la Salud Unidad Valle Dorado, Universidad Autónoma de Baja California, Ensenada 22890, BC, Mexico

**Keywords:** virucidal drugs, IBV, chickens, Coronaviridae, silver nanoparticles, artificial ribonuclease, monoglyceride lauryl acid

## Abstract

Infectious bronchitis (IB) of chickens is a highly contagious disease characterized by damage of the respiratory system and reproductive organs in young animals caused by a virus of the genus Gamma coronavirus. The condition of the respiratory system caused by the IB virus in chickens has many similarities with the pathology of the respiratory system caused by SARS-CoV-2 in humans. The effectiveness of virucidal drugs (Argovit, Triviron, Ecocid, and lauric acid monoglyceride) was tested on chickens inoculated with a tenfold dose of a vaccine strain based on the attenuated virus H120 against IB of chickens. On the 6th day after inoculation, inflammatory changes in the intestines, lungs, and thymus were observed in the control group. The experimental groups were characterized by less pronounced inflammatory reactions and a lower proportion of thymus and lung probes containing genomic IB virus RNA. Since the virucidal activity of four orally administrated formulations was possible only in the intestine, the experimental data indirectly confirmed the hypothesis of the possibility of the predominant accumulation of coronaviruses in the intestine and subsequent lung damage due to the hematogenous redistribution of viral particles and IBV antigens. It was suggested that other coronaviruses including SARS-CoV-2 can implement a similar mechanism.

## 1. Introduction

Infectious bronchitis (IB) of chickens is a highly contagious disease characterized by damage to chickens’ respiratory and reproductive organs [1,2,3,4,5]. Diseases of the respiratory system caused by the infectious bronchitis virus (IBV) [5] have common features with the pathology of the respiratory system caused by SARS-CoV-2 in humans [6]. IB and SARS-CoV-2 viruses have many similarities: both are low copy coronavirus, both have a lipid envelope, and for both, the immune system reaction contributes significantly to the damage. These give the basis to believe that the mechanisms of action of these two viruses are similar.

Bacterial viruses (bacteriophages) are a very convenient model for studying various drug virucidal activities. Specifically, bacteriophage φ6 belonging to the Cystoviridae family is a promising model. These phage particles have a spherical or icosahedral shape and dsRNA type of nucleic acid. The presence of capsids in its composition makes it an effective model for testing the activity of virucidals and disinfectants against enveloped viruses, including coronaviruses. The virucidal activity of substances determined with the φ6 bacteriophage model allows the estimation of their activity against various viruses containing lipids in their capsid, such as herpesviruses 1 and 2 and coronavirus SARS-CoV-2, which infect eukaryotic cells [7,8,9]. The φ6 bacteriophage infects *P. phaseolicola* plant pathogen bacteria. Consequently, the advantage of working experimentally with a φ6 bacteriophage and plant pathogenic bacteria is that neither the phage nor the bacteria are pathogenic to humans, which minimizes the requirements for the laboratory biosafety level. Some research groups successfully applied the φ6 bacteriophage as a model for the SARS-CoV-2 virus [10,11]. 

Drugs possessing virucidal activity in the small intestine can be effective against viral infections in other tissues. Clinical observations showed the effectiveness of Ecocid, Triviron, Argovit, and lauric acid monoglyceride (C12) against viral mal-absorption in poultry farms (flavivirus, astroviral etiology). Ecocid shows high antibacterial and virucidal activity combined with low toxicity and stability in a living organism, making it a promising alternative as a topical virucidal agent [12] and a disinfectant that prevents the horizontal transfer of antibiotic resistance genes [13]. Triviron virucidal formulation began to be used in veterinary medicine relatively recently [14,15]. The mechanism of action has no analogs and belongs to a new pharmacological group of synthetic ribonucleases [2,16]. Silver nanoparticles [8,17,18,19,20] including Argovit AgNPs [21,22,23,24] have recently shown virucidal effects against some viruses, and are therefore also potentially effective against the SARS-CoV-2 virus. Lauric acid monoglyceride (Monolaurin) is known to inactivate lipid-coated viruses by binding to the lipid–protein envelope of the virus, thereby preventing it from attaching and entering host cells, making infection and replication impossible [25]. Other studies show that Monolaurin disintegrates the protective viral envelope, killing the virus [26,27]. Monolaurin has been studied to inactivate many pathogens, including the Herpes simplex virus [25,26,27,28]. 

The present study aimed to screen the virucidal activity of the four formulations mentioned above on in vitro (bacteriophage φ6) and in vivo (IB of chickens) models for SARS-CoV-2 infection.

## 2. Materials and Methods

### 2.1. Formulations

Argovit 1% is an aqueous suspension of highly dispersed silver nanoparticles (0.6 mg/mL of metallic silver) stabilized with polyvinylpyrrolidone, produced by the Center of Investigation and Production “Vector-Vita”, Novosibirsk, Russia. Argovit™ is a stable suspension in water with an AgNP concentration of 200 mg/mL (20% *w*/*w*). The metallic Ag (content 1.2% *w*/*w*) is stabilized with polyvinylpyrrolidone (PVP 12.6 ± 2.7 kDa, content 18.8%) of AgNP total weight. The remaining 80% of the weight is distilled water. AgNPs have a spheroidal shape with a size varying from 1 to 90 nm and an average diameter of 35 ± 12 nm. The hydrodynamic diameter is 70 nm, the ζ potential is −15 mV, and a plasmonic resonance peak is registered at 420 nm [29]. Triviron (0.03% synthetic ribonuclease (1,5-bis-[N, N-1- (4-tetradecyl) diazoniabicyclo [2.2.2] octyl] pentane tetrobromide) was produced by «Trionisvet» Ltd., Korolev, Russia. Ecocid (0.05%), produced by Krka, d. d., Novo Mesto, Slovenia, contains a triple salt of potassium peroxomonosulfate (50%), as well as auxiliary substances: surfactants (sodium dodecylbenzene sulfonate), organic acids (malic, sulfamic), inorganic buffer systems (sodium chloride and sodium polyphosphate), Azo Diestaff dye, and Citron as an aromatic additive with a lemon scent. Used in our work, Ecocid, Triviron, and Argovit are certified and are commercially produced. Lauric acid monoglyceride (0.005%) was synthesized and kindly provided by Dr. Fomenko Vladislav from institute N.N. Vorozhtsov, Novosibirsk Institute of Organic Chemistry of SB RAS. 

### 2.2. Bacteriophage φ6 In Vitro Model

Bacteriophage φ6 (belonging to the *Cystoviridae* family) was used as an in vitro model to assess the formulations’ virucidal activity. A *Pseudomonas phaseolicola* cell culture was used to determine phage particle activity. The tested preparations were incubated with a bacteriophage culture at room temperature at various concentrations. After 1, 5, 15, 30, and 60 min of incubation, inoculation of samples containing bacteriophages and formulations and only bacteriophage (control group) was carried out. The residual concentration of bacteriophage inoculations was determined in 10-fold dilutions. The survival rate of phage particles was determined by the method of two-layer agar (Grazia method). The studies were carried out at room temperature (26 ± 2 °C). All experiments were performed in duplicate.

### 2.3. Poultry

Cross Shaver male chickens aged 14 days and 198–210 g each were kept indoors and fed with standard granulated chicken feed. The experimental protocol involving animals was reviewed and approved by the Ethical Committee of Novosibirsk State Agrarian University of Siberian Federal Scientific Center of Agro-BioTechnologies of the Russian Academy of Sciences CM K PO 15-01-2019/No.3 of 10.03.2021.

### 2.4. Inoculum Administration

Chickens received a tenfold dose of a vaccine against IBV (from the H120 strain, live, dry) consisting of 5 lg Embryo Infectious Dose (40 per head). It was administered orally to each chicken. Experimental groups and a control group of 13 animals were formed.

### 2.5. Dosage Administration

The dosages of the preparations were as follows: Argovit and Triviron, 250 and 285 μL/animal, respectively. They were administered two times a day (in the morning and the evening) to each animal. The lauric acid monoglyceride dose was 0.1 mg/animal. Ecocide C (0.05%) dose chickens drank freely when they wanted (57 mL per day per head). For drinking water sanitation for animals, including birds, it is recommended to use 0.1% Ecocide C in water. Here, half of the recommended concentration was applied. All formulations were administered for 5 days. The animals were slaughtered on the 6th day. In Table 1, consumption per head per day for the studied formulations is summarized.

### 2.6. Histopathological Evaluation

The lung histopathological analysis was performed under an Imager D1 luminescence microscope (Zeiss) using AxioVision v 4.6.3.0 software (Zeiss, Jena, Germany). Briefly, the lung pieces (~1 mm thick) were placed in a 96-well plate filled with distilled water (150 μL per well), and 20 μL SYBRTM Green (1:1000) and 5 μL of SyproTM Ruby dye (BioRad Laboratory) were added per well. Then, they were kept for 20 min to counterstain the preparations. 

Luminescence microscopic analysis of lung pieces was carried out according to an original technique of a short period staining mode, whose principle consisted of staining a formalin-fixed biomaterial with two dyes. SYPRO Ruby intercalates proteins and glows, while SYBR Green I binds to DNA and fluoresces in orange-red and green ranges, respectively. The staining is carried out within a short period (20 min), during which dye diffusion occurs in a thin layer of cells, which avoids the intense background fluorescence of entire pieces of tissue. Thus, in contrast to histological examination, a short period staining mode is suitable for studying thin tissue layers. This mode makes it possible to visualize better epithelial and endothelial cells (for example, the inner surface of the bronchi and blood vessels and intestinal epithelium). Additionally, this mode provides a good visualization of blood capillaries, hemorrhages, and tissue saturation with hemoglobin, which is accompanied by a sharp decrease in fluorescence intensity. The difference of this luminescence microscopic mode from confocal microscopy lies in the lower price of the used equipment, faster staining process, more detailed observations of epithelium surfaces, and the influence of the diffusion process microscopic picture.

### 2.7. qRT-PCR

RNA was isolated from internal organs using silica columns, with preliminary lysis of cells with guanidine isothiocyanate. The copy number of the IBV was assessed by qRT-PCR [14].

## 3. Results

### 3.1. Verification of the Virucidal Action with Bacteriophage φ6

Experiments with a virus model (bacteriophage φ6) were conducted as a preliminary test to estimate active doses of the studied formulations. Triviron’s effective concentration was ≥0.003% (30 μg/mL) for 1 min. At this point, the formulation completely suppressed the activity of phage particles (Table 2). Ecocid, at a concentration of 0.05% (500 μg/mL), completely suppressed viral activity in vitro within a minute, while Argovit, at a concentration of 0.00001% (0.10 μg/mL) of metallic silver, inactivated viral particles at ≥ 15 min of incubation. Lauric acid monoglyceride practically did not show a decrease in bacteriophage φ6 concentration for 60 min. So, experiments in vitro on the bacteriophage φ6 model demonstrated that among the four studied formulations at used concentrations, the virucidal activity decreases in the order: Ecocid ≈ Triviron > Argovit >> lauric acid monoglyceride. 

The presence of capsids in the composition of the bacteriophage φ6 makes it an effective model for testing the activity of virucidal and disinfecting drugs against enveloped viruses, including coronaviruses. As follows from the obtained results (Table 1), Ecocid, Triviron, and Argovit in the used concentrations in in vitro experiments suppressed the activity of the enveloped virus, whereas lauric acid monoglyceride was practically inactive.

### 3.2. Postmortem Examination 

**Figure 1:**
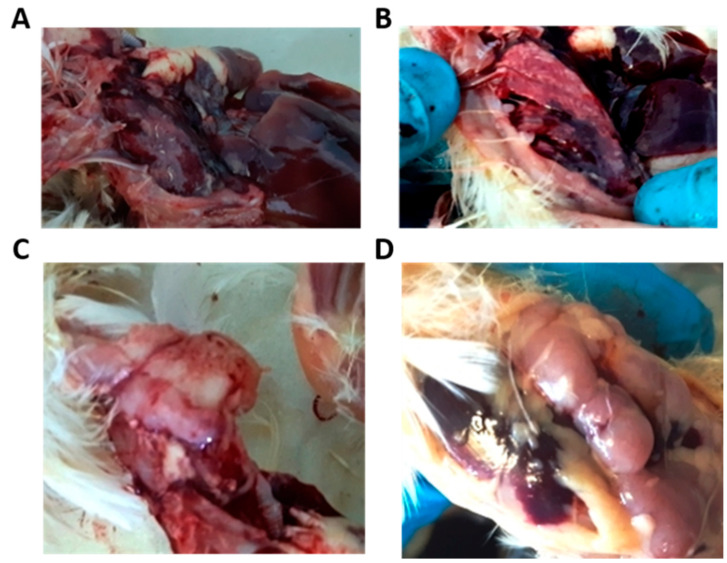
Macroscopic observations in lungs and thymus of control and experimental groups. Pneumonia signs were observed in the control group lungs (**A**) and experimental group lungs (**B**). Thymus inflammation in the control group (**C**) and thymus without noticeable changes in the experimental group (**D**).

After the autopsy, the characteristic changes in the thymus (the IBV affects the medullar zone of the thymus) and lung were noted (Figure 1). The lungs were hyperemic, edematous, and sometimes triangular and diamond-shaped lesions were observed, indicating hematogenous drift of the infectious agent to these zones. The small intestine also showed hyperemia. The large intestine was unchanged. The kidneys were not inflamed. In most experimental groups, changes in the intestines, lungs, and thymus were completely absent or less pronounced than in the control group. The most severe lesions were observed in the control and then in the lauric acid monoglyceride-treated group organs.

Inflammatory changes in the intestines were observed in all groups, but these changes were lesser in the Ecocid and Triviron groups. Lung lesions relative to the control group were less pronounced in the Triviron-treated group. The smallest number of inflammatory reactions in the thymus was observed in poultry after Ecocid and Triviron application. So, the postmortem examination showed that the inflammatory responses in the studied groups increase in the following order: Triviron < Ecocid < Argovit < lauric acid monoglyceride < control.

### 3.3. Lung Luminescence Microscopy Histopathological Study

#### 3.3.1. The Control Group

**Figure 2:**
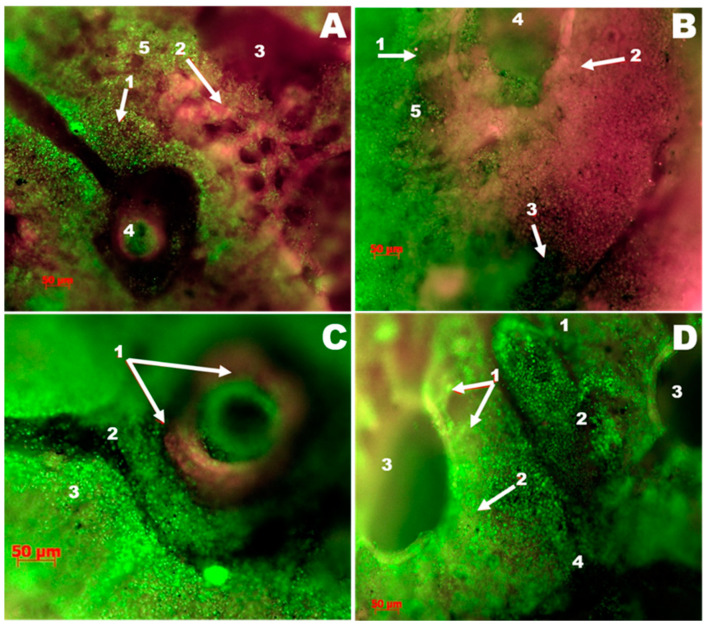
Luminescence microscopy photograph. Lungs and parabronchi of the control group stained with SYBR Green and Sypro Ruby (×50). (**A**) Interstitial inflammation in the lungs: 1—infiltrates, 2—air capillaries, 3—parabronchus, 4—blood vessel, 5—areas of the parabronchial segment with a decrease in the lumen of the air capillaries due to infiltration. (**B**) Focal changes in the lung: 1—inflammatory infiltration of the parabronchial segment, 2—saturation with serous-catarrhal exudate of the lung parenchyma and loss of the lumen of the airway capillaries, 3—hemorrhage, 4—the lumen of the parabronchus, 5—preserved air capillaries. (**C**) Vasculitis in the vessels: 1—fibrinoid inflammation of the vessel wall (artery), 2—suppression of fluorescence at the site of tissue imbibition by hemoglobin, 3—increased density of cells associated with inflammatory infiltration of the interstitium of the lung. (**D**) Inflammatory changes in the parabronchi: 1—air capillaries that have retained the lumen, 2—air capillaries that have lost their lumen due to inflammatory infiltration, 3—parabronchi, 4—hemorrhages around the affected blood vessel.

The control group is predominantly characterized by interstitial pneumonia in focal lesions involving the groups of segments of the parabronchus. In this case, some parabronchus does not present pathologic morphological changes, and others were excluded from the gas exchange, which leads to a decrease in overall gas exchange. The protein fluoresces in red; a reduction is due to infiltrative processes. An increase in luminosity can be associated with exudative processes and respiratory epithelium desquamation. Hemorrhages were found (Figure 2A). A narrowing of the air capillaries further develops the inflammatory process due to thickening the interstitium and lamina propria associated with inflammatory infiltration (Figure 2B). More severe lesions are accompanied by the accumulation of serous exudate and total overlap of the lumens of the airway capillaries; exudate rich in cells accumulates in the parabronchus (Figure 2B), and diffuse hemorrhages appear. The fact that the lesion of the pulmonary parenchyma is topologically associated with hematogenous drift of the pathogen can be confirmed by identifying signs of vasculitis and perivascular hemorrhage infiltrates (Figure 2C). Another criterion for the primary involvement of blood vessels in the pathological process (from the pulmonary artery side) is the absence of a uniform radial lesion of the parabronchial segments (Figure 2D). As shown in Figure 2D, there is an overlap of the air capillary lumens on the pulmonary parenchyma areas adjacent to the site of interstitial inflammation. Opposing areas of the parabronchial segments present a well preserved network of air capillaries. 

#### 3.3.2. The Group Treated with Argovit

**Figure 3:**
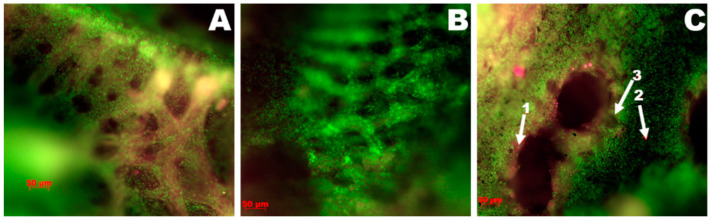
Luminescence microscopy photograph of lung preparations of the group treated with Argovit, stained with SYBR Green and Sypro Ruby (×50). (**A**,**B**) No inflammatory changes were observed. (**C**) The bronchus lumen: 1—the parabronchus wall, 2—the air capillary mouth, 3—the lung parenchyma in a state of vascular hyperemia.

A comparative analysis of the results obtained in luminescence microscopy histopathological study revealed that the lungs were less affected in the group receiving the Argovit formulation. So, the Argovit-treated group was chosen for comparative analysis with the control group. A change interval from the complete absence of signs of inflammation to significant changes like the ones presented in Figure 2 was observed. Thus, Figure 3A,B show photographs of two lung samples characterized by a complete lack of inflammatory changes. The histoarchitecture of the respiratory surfaces of the parabronchus is not disturbed; there are no inflammatory infiltrates and hemorrhages. In contrast, lung changes compared to the control group in some individuals were observed (Figure 3C).

#### 3.3.3. IBV in Chickens with RT-PCR 

##### Intestine

**Figure 4:**
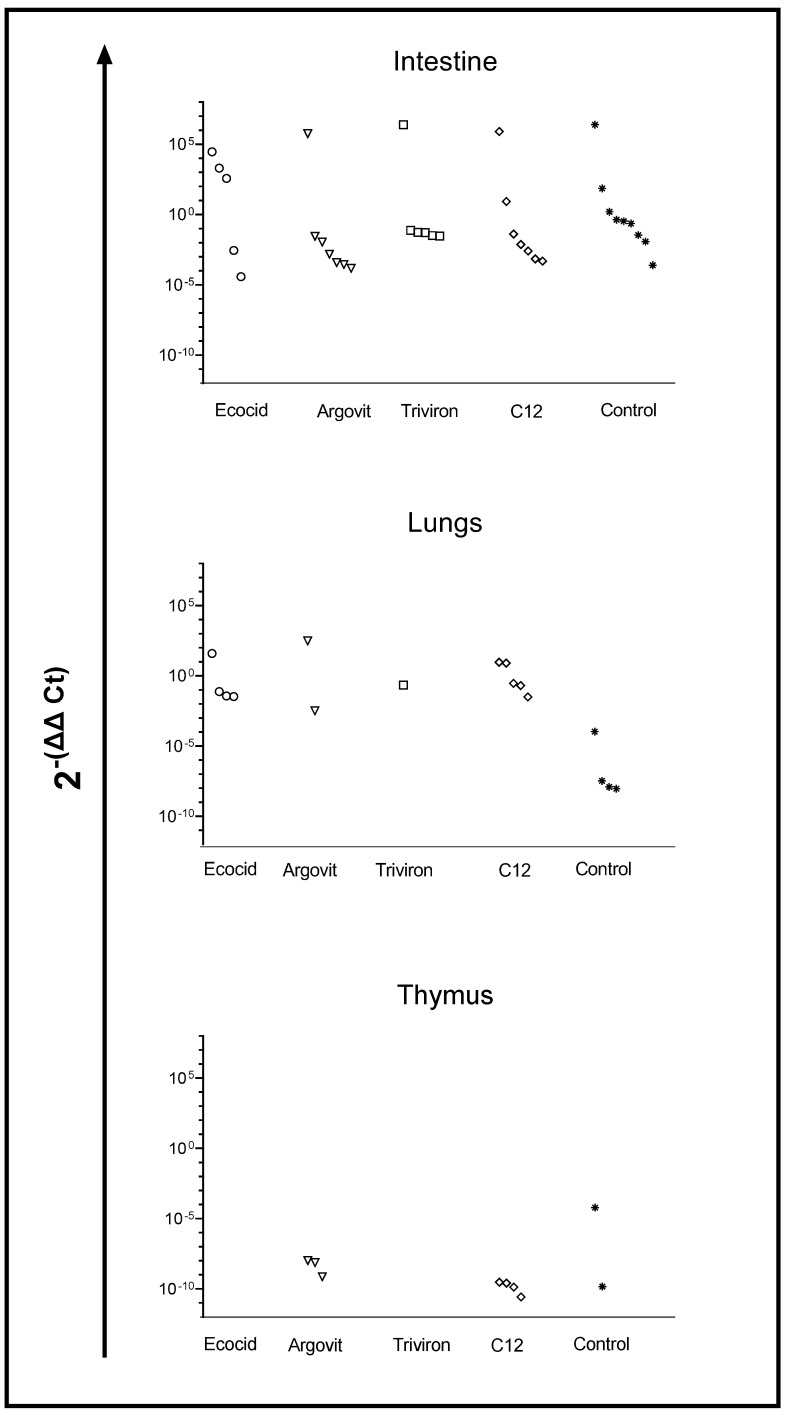
The concentration of IBV in the intestine, lungs, and thymus in chickens of the experimental and control groups. Data are presented in 2^−(ΔΔCt)^ units, which reflect the concentration of IBV from chickens treated with Ecocid (O), Argovit (∇), Trivirion (□), C12 (◊), Control (*). Every group contains data for 13 chickens.

**Figure 5:**
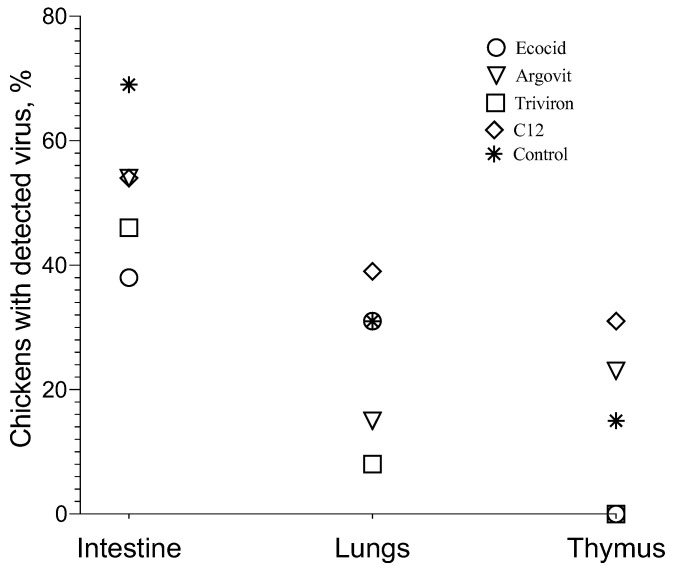
The percentage of chickens with virus detected in intestine, lungs, and thymus for all studied groups.

As shown in Figure 4, the virus concentration detected in the intestine in all studied groups varied in 8–10 orders of magnitude range, changing from 0.000038 to 2,482,450. At the same time, it is important to mention that the virus was detected in ≤69% of poultry intestines of each group. In the control group, viral concentration was detected in 69% of poultry intestines and varied 10 orders of magnitude from 0.000245 to 2,482,450 (Figure 4 and Figure 5). After formulation treatments, virus concentration in the intestine changed in 8–9 orders (9 orders for Ecocid, Argovit, and C12 and 8 orders for Triviron), and poultry percentage with the detected virus was 38%, 54%, 54%, and 46%, respectively (Figure 4 and Figure 5). 

##### Lungs

Virus concentration in lungs varied 0–6 orders of magnitude: 4, 6, 0, 3, and 4 for Ecocid, Argovit, Triviron, C12, and the control group, respectively (Figure 4). For Ecocid, Argovit, Triviron, C12, and the control group, the number of chickens with the detected virus was four, two, one, five, and four among thirteen chickens in every group, which corresponds to 31, 15, 8, 39, and 31%, respectively (Figure 4 and Figure 5). 

##### Thymus

After treatment with Ecocid and Triviron, the virus was not detected by PCR. Virus concentration in the thymus was detectable only for the Argovit, C12, and control groups (Figure 4 and Figure 5). Virus concentration varied 1-5 orders of magnitude inside these groups, being 1, 1, and 5 in the range from 7.16 × 10^−10^ to 1.03 × 10^−8^, from 2.64 × 10^−11^ to 2.95 × 10^−10^, and from 1.43 × 10^−10^ to 6.01 × 10^−5^ for Argovit, C12, and the control group, respectively (Figure 4). The virus was detected on 23, 31, and 15% of poultry thymus, respectively (Figure 4 and Figure 5).

## 4. Discussion

The results showed that treatment with all four studied formulations decreased the poultry percentage with virus detected in the intestine compared to the control group. The percentage of chickens in which the virus was detected on intestines was considerably higher in the control group (69%) than in experimental groups, being 38, 54, 46, and 54% for Ecocid, Argovit, Triviron, and C12, respectively (Figure 4 and Figure 5). The variation in virus concentration in the control group (11 orders of magnitude) was slightly higher than in experimental groups (8–9 orders of magnitude). 

The investigation of virus concentration in the lungs (Figure 4 and Figure 5) showed a decrease in the fraction of chickens with detectable virus concentration, virus concentration, and an interval of variation in detected virus concentration in each group compared with the intestine. The fraction of chickens with detectable virus concentration in the lungs was lower than in the intestine, being 31% < 38% for Ecocid, 15% < 54% for Argovit, 8% < 46% for Triviron, 39% < 54% for C12, and 31% < 69% for the control group (Figure 5). These results showed that virus RNA in the lungs was detected on a minimum fraction of chickens for Triviron (8%) and Argovit (15%). These results are consistent with histopathological studies, where Argovit showed minimal lesions in epithelial and endothelial cells in the lungs (Figure 2). Maximum virus concentrations measured in the lungs were less than in the intestine for all studied groups. These concentrations decreased by 3, 7, 5, and 10 orders of magnitude for Ecocid, Triviron, C12, and the control group, respectively. However, the concentration only decreased by half for Argovit. The virus concentrations in lungs varied in all groups between 0 and 6 orders of magnitude: 4, 6, 0, 3, and 4 for Ecocid, Argovit, Triviron, C12, and the control, respectively. 

Intestine virus concentrations varied in all groups between 8 and 11 orders of magnitude. This difference could indicate that intestinal tissue has conditions promoting viral replication, while lung tissue is more restrictive to viral replication. Unexpectedly, it was observed that virus concentrations in the lungs in the control group were much lower (between 9.19 × 10^−9^ and 1.07 × 10^−4^) than in experimental groups (between 3.3 × 10^−3^ and 3.1 × 10^2^). The explanation of this effect will need further studies. In contrast, thymus viral concentration was very low (Figure 5), and for Ecocid and Triviron, viral RNA was undetectable using qRT-PCR. For Argovit, C12, and the control group, most values were between 2.6 × 10^−11^ and 1.0 × 10^−8^. Additionally, only one data point for the control group was higher (6.0 × 10^−5^). This might be related to the fact that the thymus belongs to the immune system and, therefore, it highly restricts viral replication and/or eliminates viral particles. 

All the above results demonstrate that all four studied formulations demonstrated virucidal activity against IB virus. Argovit, Triviron, and Ecocid showed some advantages compared with C12. However, in general, the difference in the virucidal activity of the four formulations was not significant. This work is only a first approximation of using these formulations against IBV, and further studies are needed to clarify the potential use of these formulations.

Obtained data showed that on the 6th day after IBV injection, the virus concentration was significantly higher in the intestine than in the lungs and thymus in all groups. The period of six days after inoculation was adequate time for the dissemination of the virus to all organs by blood circulation. Supposing that the intestine was not the organ with optimal conditions (among the three studied organs) for virus replication, after 6 days, the concentration of the virus would be maximal in other organs (if they have optimal conditions for replication), for example, in the lungs. However, this was not observed experimentally. Instead, the highest viral concentration was observed in the intestine. Hence, the obtained results suggest that the intestine is the organ with the highest cell secretory potential, receptors, and enzyme systems suitable for the reproduction of coronavirus and the maturation of viral particles, which creates optimal conditions for primary virus replication and accumulation. It implies that, specifically, the intestine serves as a source for virus dissemination to other organs. Low virus concentrations in the lungs and thymus could indicate that viral replication is occurring relatively slowly. However, lesions observed in our experiments in the lungs might show that the virus is not eliminated quickly from the lungs because even at low concentrations it manages to cause lesions. The hypothesis about the primary role of intestinal tissue in the replication/accumulation and source of dissemination of IBV was suggested in our previous publications [1,30]. 

The obtained results suggest the following mechanism of IB virus entering the organism. Firstly, the virus enters the nasopharynx and interacts with the mucus. The nasopharynx mucus layer (a porous gel net) acts as an impenetrable physical barrier to most pathogens. However, because the pore diameter of the gel net (approximately 500 nm) is significantly larger than the IB virus diameter (50–100 nm) [4], the IB virus easily penetrates mucus pores. Once swallowed, the mucus is digested by the gastrointestinal system, and the virus starts to replicate. It is important to mention that the size of the SARS-CoV-2 virus (approximately 60–140 nm) [31] is close to the IB virus diameter (50–100 nm). 

Because IB and SARS-CoV-2 viruses are similar, the mechanism suggested above for IB virus could also be applied to the SARS-CoV-2 virus. The results of recent works confirm the validity of our hypothesis that the intestine is the principal organ for virus reproduction and accumulation, and it serves as a source of virus dissemination into other tissues. These results agree with the fact that that the highest ACE2 expression in human organisms occurs in the intestinal enterocyte brush border [32,33]. Although it was reported that ACE2 is expressed in the lung, liver, stomach, ileum, kidney, and colon, its expressing levels there are relatively low, especially in the lung. In lung AT2, this level is 4.7-fold lower than the average expression level value of ACE2-expressing cells of all 13 cell types studied in this work [32]. 

Some recently published clinical observations indicate the relevant role of the intestine in the infection caused by the SARS-CoV-2 virus, which agrees with our hypothesis. SARS-CoV-2 viral RNA can be detected in rectal swabs when nasopharyngeal testing was already negative, suggesting long-term gastrointestinal infection [34]. The recent observations showed that gastrointestinal symptoms were observed in 57% of the patients infected with SARS-CoV-2. In addition, these symptoms sometimes occurred without respiratory symptoms. Gastrointestinal manifestations are more common in patients with severe disease than in patients with non-severe disease. For approximately 48.1% of patients with severe acute respiratory syndrome COVID-19, a stool sample was positive for SARS-CoV-2 RNA virus [35]. This hypothesis [36] suggests that complications after COVID-19 could be caused by SARS-CoV-2-upregulated angiotensin II-caused disruption of mucosal barriers with following microbial and/or lipopolysaccharide movement from the gastrointestinal tract into various tissues. 

Coronavirus modulates autophagy or its components for its benefit, primarily the autophagosomes used as replication and transcriptional niches. The IBV induces the formation of autophagosomes via the MAPK/ERK1/2 pathway dependent on Atg5 [37]. However, the induction of autophagy also facilitates the activation of apoptosis by generating a platform for activating caspase-8 or depleting endogenous inhibitors of this cell death pathway [38,39], as observed on tumor cells exposed to Bortezomib, which showed apoptosis via caspase-3 activation [40,41]. 

Although MAPK/ERK1/2 pathways generally promote cell survival, certain circumstances such as DNA or oxidative damage function as pro-apoptotic signaling [42]. Oxidative stress and DNA damage have been described on several tumor cells exposed to Argovit AgNPs, promoting cell death by an intrinsic apoptotic pathway [43,44]. Thus, the apoptotic pathway could be activated on cells harboring IBV due to the virus-promoted autophagosome increase and redox damage promoted by the presence of AgNPs, leading to cell death and the inhibition of virus proliferation. This fact could explain the viral titer decrease observed in the lungs after the administration of silver nanoparticles.

Furthermore, the absence of inflammatory infiltrates or hemorrhages observed in the lungs treated with AgNPs could be associated with the selective cytotoxic effect that should be kept only on those cells with increased autophagosomes, which are more susceptible to oxidative damage than non-infected cells. The above-mentioned cytotoxic selectivity was already observed on tumor and non-tumor cells exposed to AgNPs, where non-transformed cells showed no oxidative damage compared with tumoral cells [45].

It is also worth mentioning that recently published works describe the first experimental data on infection prevention with SARS-CoV-2 in humans at least for two formulations studied here (Argovit and C12). For example, recently, Argovit was studied in vitro and in vivo to prevent SARS-CoV-2 infection in health workers. The inhibitory effect of AgNPs in SARS-CoV-2 NL/2020 strain replication in cultured Vero E6 cells was confirmed [46]. A randomized study (with 114 and 117 participants in experimental and control groups, respectively) demonstrated that mouthwash and nose rinse with 1% Argovit-C reduced the SARS-CoV-2 infection rate 16 times in healthcare personnel attending on average 169 patients with COVID-19 per week per person [47]. It was demonstrated that the silver nanocluster/silica composite coating deposited on facial masks possessed a virucidal effect against SARS-CoV-2 [29]. The investigation of blood serum samples collected from 51 healthcare workers of an Italian COVID-19 hospital showed that a higher concentration of C12 was observed in protected workers compared with those infected with SARS-CoV-2 [46]. The authors suggested a potential defensive role of monolaurin against SARS-CoV-2 infection. They offered a randomized controlled trial of monolaurin supplements to confirm these observational findings before any therapeutic recommendations can be made.

Hence, the results of our study showed that it is vital to consider the prospects for combating coronavirus infections, including COVID-19, using virucidal drugs. Virucidal drugs are substances causing the inactivation of viral particles, thereby limiting the infection of new cells and preventing the damage of cells and body tissues. Silver nanoparticles should also be included in this class of drugs. Unlike classical antiviral drugs, for example, inhibitors of the activity of RNA-dependent RNA polymerase of coronaviruses (Areplivir, Remdesivir, etc.), virucidal drugs are not required to penetrate the cell to exert their virucidal effect. For this reason, the dosage of virucidal medicines may be less. Therefore, their toxic effect may be significantly less due to poor transport into cells (which reduces the risks of metabolic disorders).

One feature of the pharmacokinetics of many virucidal drugs is poor penetration through mucous barriers. However, the inactivation of viral particles on the surface of mucous membranes (intestines, nasopharynx) with virucidal medications, combined with an intensive self-cleaning of the surfaces of mucous membranes, creates good prospects for limiting the entry of the SARS-CoV-2 virus into the blood and lymphatic vessels, followed by hematogenous and lymphomatous transport to the pulmonary circulation (lungs). The intensity of biosynthetic processes (such as virus replication) in the epithelium of mucous membranes is extremely high. Limiting the reproduction of the virus in the nasopharynx and intestine can have a systemic effect on the development of COVID-19.

## 5. Conclusions

Four formulations (Ecocid, Triviron, Argovit, and lauric acid monoglyceride) possessing virucidal activity in the small intestine showed potential against chicken infectious bronchitis virus. Based on the obtained results, our hypothesis is that the transmission of IB virus in chickens occurs not through the respiratory system but through the intestine, where more RNA was determined. Then, from there, it is disseminated to other organs, including the lungs. To the best of our knowledge, our group is the first to propose such a route of infection for the IB virus.

Considering that IB is proposed as a model for SARS-CoV-2, because both viruses are low-copy coronaviruses with a lipid envelope and similar diameters, it was suggested that a similar mechanism based on primary virus replication and accumulation in the intestine could also be carried out for the SARS-CoV-2 virus. If confirmed, this paradigm may open up innovative treatments for COVID-19 and other respiratory diseases caused by a coronavirus, targeting intestinal viral load to minimize infection in other tissues.

## Figures and Tables

**Table 1:** Consumption per head per day for studied formulations.

No.	Formulation	Consumption of Active Component per Head per Day (µg)
1	Argovit C, 0.06% (metallic silver)	150
2	Triviron, 0.03%	85.5
3	Lauric acid monoglyceride (C12), 0.05%	100
4	Ecocid C, 0.05%	28,500

**Table 2:** Change in the concentration of bacteriophage φ6 after incubation with virucidal drugs.

Formulation Name and Concentration	Bacteriophage φ6 Concentration, PFU */mL					
	Initial	After Exposure Time (min).				
		1	5	15	30	60
Ecocid 0.05% (500 µg/mL)	9.6 ± 0.17 × 10^5^	0	0	0	0	0
Triviron 0.003%, (30 µg/mL)	1 ± 0.00 × 10^7^	0	0	0	0	0
Lauric acid monoglyceride (C12), 0.05%	1 ± 0.57 × 10^7^	7.4 + 1.3 × 10^7^	1.1 + 0.11 × 10^7^	2.0 + 3.3 × 10^7^	1.3 + 0.33 × 10^7^	8.9 + 6.3 × 10^6^
Argovit, 0.00001% (10 µg/mL) of metallic silver	4.2 ± 0.15 × 10^6^	3.4 + 0.63 × 10^4^	6 + 0.115 × 10^2^	0	0	0

* Plaque-forming unit (PFU).

## Data Availability

The data presented in this study are available in article.
